# Supplementary material for: Analyses of the performance of the Ebola virus disease alert management system in South Sudan: August 2018 to November 2019
Source: PLoS Negl Trop Dis. 2020 Nov 30;14(11):e0008872. doi: 10.1371/journal.pntd.0008872 (PMC7728195; doi:10.1371/journal.pntd.0008872)
Supplement: S1 Table — (PPTX) [file pntd.0008872.s002.pptx]

## Slide 1
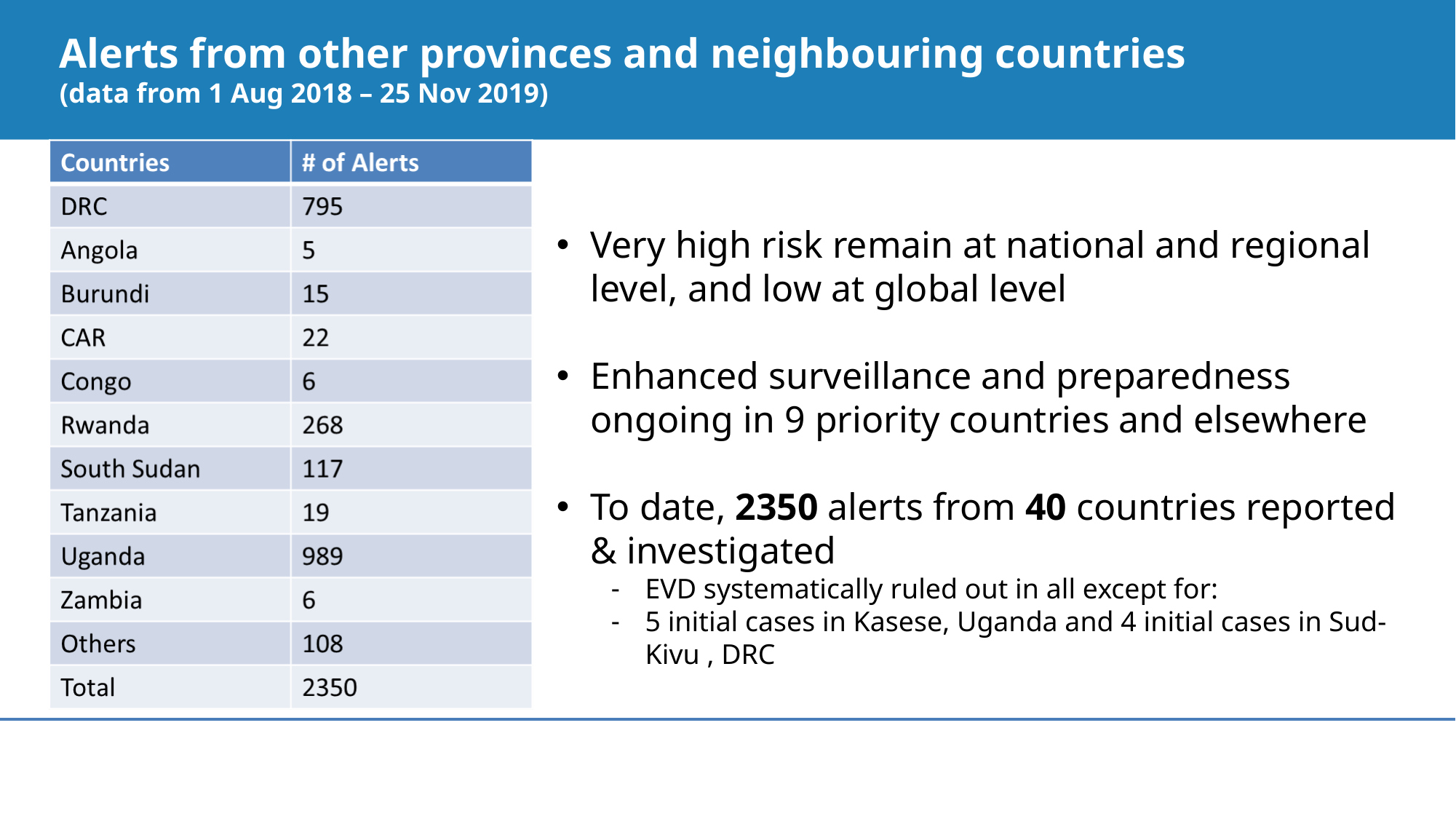

# Alerts from other provinces and neighbouring countries (data from 1 Aug 2018 – 25 Nov 2019)
Very high risk remain at national and regional level, and low at global level
Enhanced surveillance and preparedness ongoing in 9 priority countries and elsewhere
To date, 2350 alerts from 40 countries reported & investigated
EVD systematically ruled out in all except for:
5 initial cases in Kasese, Uganda and 4 initial cases in Sud-Kivu , DRC
