# Supplementary material for: Analyses of the performance of the Ebola virus disease alert management system in South Sudan: August 2018 to November 2019
Source: PLoS Negl Trop Dis. 2020 Nov 30;14(11):e0008872. doi: 10.1371/journal.pntd.0008872 (PMC7728195; doi:10.1371/journal.pntd.0008872)
Supplement: Attachment — Submitted filename: Response to Reviewers_Rev 1.docx [file pntd.0008872.s004.docx]

The Editor

*PLOS Neglected Tropical Diseases*

14 September 2020

**Dear Editor,**

As advised, we have addressed all the comments of the two reviewers. All inputs are clearly marked in track change in the attached revised manuscript for ease of reference; a clean version of the manuscript is also attached. The point-by-point changes made to the manuscript as well as responses to the comments of the reviewers are as follows:

**Reviewer #1:**

Line 258: Methodological aspects for qualitative data analysis is inadequate. How were these data analysed? Did you use any framework? How did you synthesize the data from the review of documents? Please explain in detail. You can check COREQ guidelines for complete qualitative data reporting. Link: <https://academic.oup.com/intqhc/article/19/6/349/1791966>

**The methodological aspects of the qualitative data analyses have been beefed up and an appropriate reference (reference no 20) has been included in the manuscript (lines 278-290). Subsequently, we have updated table 5 into an appropriate SWOT analyses matrix.**

Page 16: since your study is focused on descriptive epidemiology of the surveillance system for Ebola. I wonder, if you could present some of these important variables in figures that would best serve the objective of your study. For e.g.

•	source of identification of alert

•	Place of identification of alert

•	Status of alert

**These variables are now presented as Figs 2, 3 and 4 as recommended**

Table 4: I see that you are trying to compare the timelines of alert notification in days between different parts of the year. But as you earlier said, your data had a non-normal distribution, thus you used non-parametric test: Kruskal Wallis test. For non-parametric test, it is best to present median (not mean or mode) and show only p value on the side.

**The mean, mode, minimum and maximum have been deleted from table 4 and the median inserted. In line with these changes, necessary revisions have been made to the results, discussion and recommendation sections.**

Table 5: It looks like a very informative table, and I believe you have used the SWOT analysis. However, could you delineate each component, importantly, ‘opportunities’ as recommendations? That will be something interesting for other LMICs and surveillance system.

**Done; the table has been updated with each component of the SWOT analysis delineated. The methodology used for the SWOT analysis is also provided in the methods section and an appropriate reference inserted (lines 278-290). We have also proposed recommendations for addressing the weaknesses identified in the SWOT analyses (lines 501-511)**

In the conclusion, you have mentioned Boma Health initiative (BHI). I think this is the first time you have talked about BHI. I recommend authors to provide a background information about BHI in the introduction section for readers to adequately anticipate and understand what it is and how does it come into the context.

**A description of the Boma Health Initiative has been provided in the study design and setting sub-section (lines 167-173)**

Thank you for the opportunity to review the paper by Olu et al. In general, the paper is well written with contextual details concentrated in discussion section. There are some sections that needs improvement. Below, I have a few specific recommendations.

Line 197: revise and mention the capital city; is it Juba? Or were you referring somewhere else?

**This has been revised with Juba included (line 213); we also clearly stated that Juba is the capital of the country in the sub-section on study design and setting (lines 150-151)**

Line 246: Mention here who are EVD stakeholders?

**This has been corrected (lines 261 and 264)**

Line 402: IDSR and EWARN: please explain these abbreviations

**The full meaning of IDSR and EWARN have been provided at their first mention in the data analysis sub-section; we have also briefly explained both systems (lines 241-246).**

Discussion is very well written, and authors have explained in detail the interpretation and its practical relevance for South Sudan’s health system strengthening. I think the discussion will benefit from providing sub-headings for readers to easily pick up on the findings, challenges/recommendations.

**Appropriate sub-headings have been included in the discussion section (lines 389, 419, 459, 499, 513, 524 & 536)**

Explain why there are highest alerts in August, October and November?

**Explanation has been provided; please see line 449-452**

Why was there clustering of EVD alerts in Yambio?

**Explanation has been provided; please see lines 452-456**

**Reviewer #2:**

The objectives are clearly articulated with a stated testable hypothesis. The study design appropriately addresses the stated objectives. Yes, the population is clearly described and appropriate. There is a limitation (also identified by the authors) in relation to sample size but given there is no control on the number of possible EVD cases, the number of cases is adequate for a descriptive study. The authors applied the correct statistical analysis to support the conclusions. I find no concerns about ethical or regulatory requirements

**Thank you**

The analysis and the plan match the clearly presented results.

**Thank you**

The conclusions support the data presented, with the limitations of analysis well described. The in-depth discussion provide understanding on the public health relevance of the study

**Thank you**

I have addressed and identified some issues that need clarification in the body of the manuscript (uploaded). Table 1 should be presented in a landscape format

**Thank you; we have made all the changes which you pointed out in the body of the manuscript**

The manuscript makes important contributions to the detection, response control of cross-border transmission of infectious diseases. While highlighting the challenges especially in a resource constrained environment, offering practical solutions for improving response activities

**Thank you**

**Editor:**

Figure Files:

While revising your submission, please upload your figure files to the Preflight Analysis and Conversion Engine (PACE) digital diagnostic tool, <https://pacev2.apexcovantage.com>. PACE helps ensure that figures meet PLOS requirements. To use PACE, you must first register as a user. Then, login and navigate to the UPLOAD tab, where you will find detailed instructions on how to use the tool. If you encounter any issues or have any questions when using PACE, please email us at [figures@plos.org](mailto:figures@plos.org).

**All the figures have been processed using the PACE tool to ensure that they meet PLOS requirements**

Data Requirements:

Please note that, as a condition of publication, PLOS' data policy requires that you make available all data used to draw the conclusions outlined in your manuscript. Data must be deposited in an appropriate repository, included within the body of the manuscript, or uploaded as supporting information. This includes all numerical values that were used to generate graphs, histograms etc. For an example see here: <http://www.plosbiology.org/article/info%3Adoi%2F10.1371%2Fjournal.pbio.1001908#s5>.

**Done; the MS Excel database used for this study has been uploaded as supporting information**

Reproducibility:

To enhance the reproducibility of your results, PLOS recommends that you deposit laboratory protocols in protocols.io, where a protocol can be assigned its own identifier (DOI) such that it can be cited independently in the future. For instructions see <https://journals.plos.org/plosntds/s/submission-guidelines#loc-methods>

**This is not applicable to this study**

We have also made further edits to the manuscript as requested by the Editor as follow:

- **Line 389: the unpublished data has now been included as supplementary information (S1) and appropriate reference made to it in manuscript text**
- **All tables were uploaded separately to file inventory**
- **All trademark symbols have been removed from the manuscript (line 246)**

We are available to clarify the responses above and any other issues in the revised manuscript.

We look forward to your favorable consideration of the manuscript for publication and hearing from you at your earliest convenience.

**Yours sincerely,**

**Dr Olushayo Olu**
